# The Assessment of Overall Hangover Severity

**DOI:** 10.3390/jcm9030786

**Published:** 2020-03-13

**Authors:** Joris C Verster, Aurora J.A.E. van de Loo, Sarah Benson, Andrew Scholey, Ann-Kathrin Stock

**Affiliations:** 1 Division of Pharmacology, Utrecht Institute for Pharmaceutical Sciences (UIPS), Utrecht University, 3584CG Utrecht, The Netherlands; j.c.verster@uu.nl (J.C.V.); a.j.a.e.vandeloo@uu.nl (A.J.A.E.v.d.L.); 2 Institute for Risk Assessment Sciences (IRAS), Utrecht University, 3584CM Utrecht, The Netherlands; 3 Centre for Human Psychopharmacology, Swinburne University, Melbourne, VIC 3122, Australia; sarahmichellebenson@gmail.com (S.B.) andrew@scholeylab.com (A.S.); 4 Cognitive Neurophysiology, Department of Child and Adolescent Psychiatry, Faculty of Medicine, TU Dresden, Fetscherstr. 74, 01307 Dresden, Germany

**Keywords:** alcohol, hangover, symptoms, severity, measurement, scale, single item assessment

## Abstract

The aim of this study was to critically evaluate and compare the different methods to assess overall hangover severity. Currently, there are three multi-item hangover scales that are commonly used for this purpose. All of them comprise a number of hangover symptoms for which an average score is calculated. These scales were compared to a single, 1-item scale assessing overall hangover severity. The results showed that the hangover symptom scales significantly underestimate (subjective) hangover severity, as assessed with a 1-item overall hangover severity scale. A possible reason for this could be that overall hangover severity varies, depending on the frequency of occurrence of individual symptoms included in the respective scale. In contrast, it can be assumed that, when completing a 1-item overall hangover scale, the rating includes all possible hangover symptoms and their impact on cognitive and physical functioning and mood, thus better reflecting the actually experienced hangover severity. On the other hand, solely relying on hangover symptom scales may yield false positives in subjects who report not having a hangover. When the average symptom score is greater than zero, this may lead to non-hungover subjects being categorized as having a hangover, as many of the somatic and psychological hangover symptoms may also be experienced without consuming alcohol (e.g., having a headache). Taken together, the current analyses suggest that a 1-item overall hangover score is superior to hangover symptom scales in accurately assessing overall hangover severity. We therefore recommend using a 1-item overall hangover rating as primary endpoint in future hangover studies that aim to assess overall hangover severity.

## 1. Introduction

The alcohol hangover is defined as the combination of negative mental and physical symptoms which may be experienced the day after a single episode of alcohol consumption, starting when blood alcohol concentration (BAC) approaches zero [1,2]. Alcohol hangovers are typically characterized by a combination of symptoms affecting subjective mood, cognition and physical functioning [3,4,5,6]. These symptoms have been shown to negatively impact daily activities such as job performance [7] and driving [8,9,10]. The annual economic costs of alcohol hangover in terms of absenteeism and presenteeism have been estimated to be 173 billion USD for the USA [11] and 4 billion GBP for the UK [12]. Since the foundation of the Alcohol Hangover Research Group in 2010, the amount of research on the causes, consequences, treatment, and prevention of hangovers has been growing rapidly. Accurate measurement tools are essential to assess hangover severity, for example in experimental and naturalistic studies, in intervention studies examining treatment efficacy, and in survey research. Generally, they may be filled in by any drinker of all (adult) ages and in any drinking-related (research) context. There is, however, ongoing debate about which measure of hangover severity is most suitable. In this paper, we compare the three most widely used hangover symptom scales with a 1-item overall hangover severity rating, and discusses why the latter is a more reliable and useful measure to be included in future hangover research.

## 2. Characteristics of an Effective Patient-Reported Outcome Measure (PROM) 

Currently, there are no biomarkers that accurately and objectively assess hangover severity. Therefore, one has to rely on Patient-Reported Outcome Measures (PROMs). A PRO instrument is often a questionnaire/scale or single item that is directly answered by the patient, capturing the patient’s experience without interpretation of the patient’s response by a clinician or anyone else [13]. To the ensure the validity of a PRO, it is fundamental that it reliably measures the concept it is intended to measure (i.e., hangover). In the case of a multi-item scale, it is furthermore important that all of the individual items adequately contribute to the final conceptual framework of the instrument [13]. Any given scale can only be considered to have sufficient validity as a measuring tool when these conditions are fulfilled. The complex nature of alcohol hangover severity, which includes multidomain facets associated with the presence and severity of variable symptoms, and their impact on cognitive and physical functioning and mood, makes it quite challenging to develop multi-item scales that accurately assess the concept of hangover severity. Nevertheless, there are currently three hangover symptom scales available for this purpose, and they are commonly used in hangover research [14,15,16]. 

Slutske et al. [14] developed the Hangover Symptoms Scale (HSS) to assess the frequency with which drinkers experienced hangover symptoms in the last year. The scale consists of 13 items including “felt extremely thirsty or dehydrated”, “felt more tired than usual”, “experienced a headache”, “felt very nauseous”, “vomited”, “felt very weak”, “had difficulty concentrating”, “more sensitive to light and sound than usual”, “sweated more than usual”, “had a lot of trouble sleeping”, “was anxious”, “felt depressed”, and “experienced trembling or shaking”. Items can be scored either dichotomously (experienced the symptom, or not), or on a 5-point scale from “never”, “2 times or less” (once or twice per year), “3–11 times” (more than once or twice, but less than once per week), “12–51 times” (more than once a month, but not every week), and “52 times or more” (once per week or more frequently). It is important to underline that the original HSS outcome is a frequency measure. The scale has, however, been modified and used to assess hangover severity, for example by using the same items but changing the item scoring into a symptom rating ranging from 0 (absent) to 10 (extreme) [3]. 

Rohsenow et al. [15] developed the Acute Hangover Scale (AHS) to assess hangover severity. The scale consists of nine items including “hangover”, “thirsty”, “tired”, “headache”, “dizziness/faintness”, “loss of appetite”, “stomachache”, “nausea”, and “heart racing”, which are rated on a scale ranging from 0 to 7. The anchors of the scale are “none” (score of 0), “mild” (score of 1), “moderate” (score of 4), and “incapacitating” (score of 7). Overall hangover severity is computed by calculating the average score across the AHS nine items. 

The third scale, developed by Penning et al. [16], is the Alcohol Hangover Severity Scale (AHSS). It consists of 12 items, including “fatigue (being tired)”, “clumsiness”, “dizziness”, “apathy”, “sweating”, “shivering”, “confusion”, “stomach pain”, “nausea”, “concentration problems”, “heart pounding”, and “thirst”. Symptom severity for each item can be rated on a scale ranging from 0 (absent) to 10 (extreme). Overall hangover severity is the average score across the 12 items. 

The three hangover symptom scales each present with some shortcomings, and their limitations are discussed in detail elsewhere [16]. For example, scales do not always include true hangover symptoms (e.g., the HSS includes the item “trouble sleeping”, which is experienced before the start of the hangover state). The AHS includes the item “hangover”, but it is usually not advised to include an item that is identical to the overall concept that one aims to measure with a multi-item scale. Finally, the AHSS does not include the item headache, even though this is a frequently reported hangover symptom. Notwithstanding these limitations, the three hangover symptom scales are currently the most frequently used scales to assess hangover severity. Other researchers such as Hogewoning et al. [17] have been using an extended symptom listing, including all symptoms of the three hangover symptom scales.

Alternatively, overall hangover severity may be assessed with a single item rated on a scale ranging from absent (0) to extreme (10). This 1-item score is hypothesized to encompass all symptoms experienced by the drinker, including their relative impact on daily activities and mood.

## 3. Comparing Overall Hangover Severity Outcomes of the Different Assessment Methods

When using multi-item instruments, it is important that all items are relevant to the concept under investigation (i.e., alcohol hangover). If irrelevant items were included (e.g., symptoms that are seldomly reported), this would result in an overall hangover severity score that would be biased towards zero, and would therefore tend to underestimate, or even not show, the effects of treatment in intervention studies. In extreme cases, this might even lead to the wrong conclusion that a treatment is ineffective [13]. 

Penning et al. [3] examined the scientific literature and identified 47 hangover symptoms. However, the AHSS, HSS and AHS each comprise a different selection of these symptoms. This selection of symptoms may have a significant, and potentially biasing effect on the aggregate rating of overall hangover severity. The discrepancy between aggregate symptom scores and 1-item overall hangover severity assessments has been demonstrated previously by Penning et al. [16]. In that study, 947 subjects (Mean (SD) age of 21.1 (2.3) years old, 46 % men) rated the presence and severity of 23 hangover symptoms on a scale ranging from 0 (absent) to 10 (extreme). In addition, overall hangover severity was assessed. Further evaluation of the data showed that mean (SD) scores on a modified HSS and the AHSS were 3.6 (1.4) and 3.7 (1.7), respectively. Of note, the mean (SD) severity score on the 1-item hangover scale, i.e., 5.7 (2.2), was significantly higher (*p* < 0.0001) than both the modified HSS and the AHSS hangover score. These observations suggest that hangover severity scores based on aggregate symptom scores significantly underestimate the true hangover severity assessed with a single overall severity item.

There are three important reasons why aggregate symptom scores may deviate from the true hangover effect. These are related to (1) the relative presence and severity of hangover symptoms, (2) the impact of the experienced symptoms on cognitive functioning, physical activities, and mood, and (3) the fact that several assessed symptoms are also experienced without having a hangover, or even without consuming alcohol at all. These issues are discussed further in the next sections. 

## 4. Presence and Severity of Hangover Symptoms

**Figure 1:**
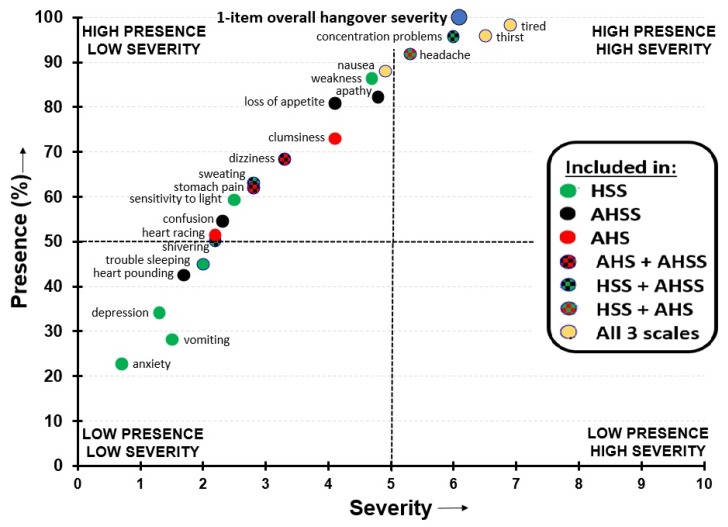
Presence and severity of symptoms included in hangover symptom scales. Data from *n* = 1837 social drinkers who reported on their latest past month hangover [4]. Note: “sensitivity to sound” was not assessed. Abbreviations: HSS = Hangover Symptoms Scale, AHSS = Alcohol Hangover Severity Scale, AHS = Acute Hangover Scale.

The occurrence and severity may differ significantly between symptoms experienced in the hangover state. This is illustrated by evaluating the data by Van Schrojenstein Lantman et al. [4], which is depicted in Figure 1. This study surveyed *n* = 1837 social drinkers who reported overall hangover severity and the presence and severity of individual hangover symptoms experienced in their last hangover in the past month, and rated this on a scale ranging from 0 (absent) to 10 (extreme). On this occasion, they reported consuming a mean (SD) of 12.6 (5.5) alcoholic drinks, corresponding to an estimated peak BAC of 0.19 (0.1) %. Figure 1 shows that both the frequency of occurrence and severity differed considerably between individual hangover symptoms. Most individual symptom scores are lower than the 1-item overall hangover severity score, suggesting that a symptom average score will underestimate overall hangover severity. As the three hangover scales comprise different hangover symptoms, it is understandable that the aggregate symptom scores of these scales differ from each other. Several symptoms, such as depression and anxiety (both low frequency/low severity), have a limited contribution to the aggregate scale score, whereas other symptoms, such as concentration problems and being tired (both high frequency/high severity symptoms), have a large contribution to the aggregate score. Including low frequency/low severity symptoms or excluding high frequency/high severity symptoms results in an underestimation of the “true” overall hangover severity. It is evident from Figure 1 that the HSS, especially, contains several low frequency/low presence items. Therefore, HSS scores likely underestimate the true hangover severity to a greater extent than the AHS and AHSS. 

A similar variability in the presence and severity of hangover symptoms was recently reported by Van Lawick van Pabst et al. [18]. Omitting relevant items from a scale can have a significant impact on the overall rating of hangover severity. An example from the dataset of Van Schrojenstein Lantman is the item “sleepiness”, which is not included in any of the three hangover scales. Sleepiness was reported by 97.1% of participants and its severity was rated as 6.5 out of 10 (extreme). It can be assumed that when completing a single item overall hangover severity item, the subject’s rating is influenced by all symptoms and feelings the subject experiences during the hangover state. Therefore, aggregate scale scores of a limited number of symptoms are very likely to underestimate the true overall hangover severity. 

## 5. Negative Impact of Hangover Symptoms

When judging overall hangover severity, it is likely that drinkers will take into account to what extent all experienced individual hangover symptoms negatively affect their cognitive functioning, physical activities, and mood. Symptoms with the largest negative impact on these domains are not necessarily those symptoms that have the highest severity scores. There is also no relationship between the impact symptoms may have and the relative frequency of occurrence in the overall drinking population. For example, heart racing can be a very disturbing effect and have a significant impact on mood. However, the symptom is not frequently reported. Alternatively, severity scores and the presence of ratings of being thirsty are usually high, while effects on cognitive functioning, physical activities, and mood are virtually absent. 

**Figure 2:**
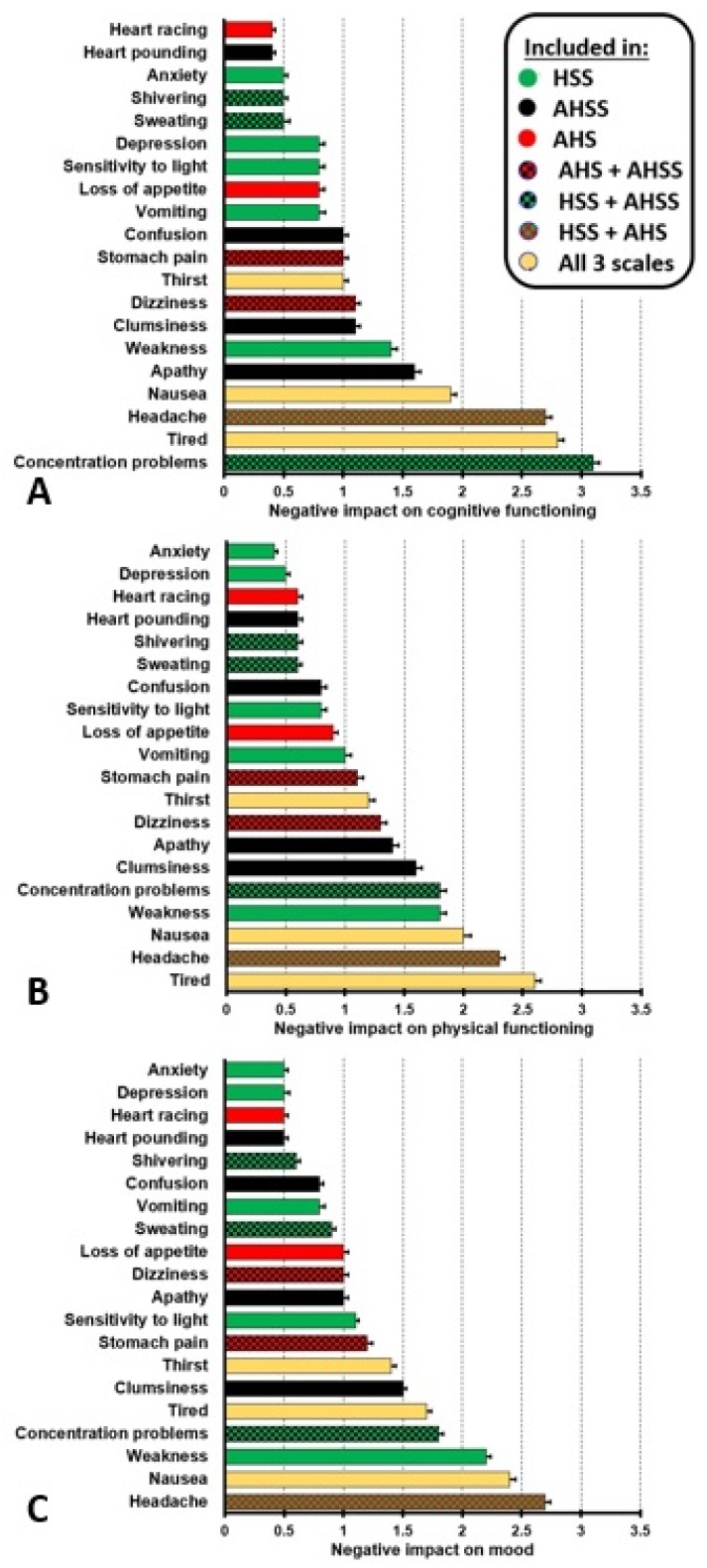
Impact of hangover symptoms on cognitive and physical functioning and mood. Negative impact of hangover symptoms cognitive functioning (**A**), physical functioning (**B**), and mood (**C**) was rated on scales ranging from 0 (absent) to 5 (extreme). Note: “sensitivity to sound” was not assessed and “trouble sleeping” was excluded as not being a true hangover symptom. Data from reference [4].

Van Schrojenstein Lantman et al. [4] also examined the impact of experiencing hangover symptoms on cognitive and physical functioning, and mood in *n* = 1837 social drinkers who reported on their last hangover experience in the past month. Negative impact of hangover symptoms on cognitive and physical functioning, and mood was rated on scales ranging from 0 (absent) to 5 (extreme). The results are summarized in Figure 2.

It is evident from Figure 2, that there are clear differences regarding the extent that hangover symptoms have an impact on cognition, physical activities, and mood. Therefore, the specific items that are included in a hangover symptom scale determine to what degree the true overall hangover effect is accurately reflected in an aggregate score (especially if no item weights are used during the formation of the composite score). As the hangover symptom scales do not include all imaginable hangover symptoms, while at the same time providing items that may not apply to a given participant, they will likely underestimate the “true” overall impact of hangover symptoms on cognition, physical activities, and mood. This can again be illustrated by considering the hangover symptom “sleepiness”. Although not included in any of the three hangover symptom scales, Van Schrojenstein Lantman et al. [4] found that sleepiness was reported by 97.1% of drinkers. The mean (SD) impact scores for sleepiness were 2.7 (1.7) for cognitive functioning, 2.5 (1.7) for physical functioning, and 2.4 (1.6) for mood. If this symptom was incorporated in a scale, it would very likely have influenced the aggregate impact score. 

## 6. Symptoms May also be Present without a Hangover or Alcohol Consumption

**Figure 3:**
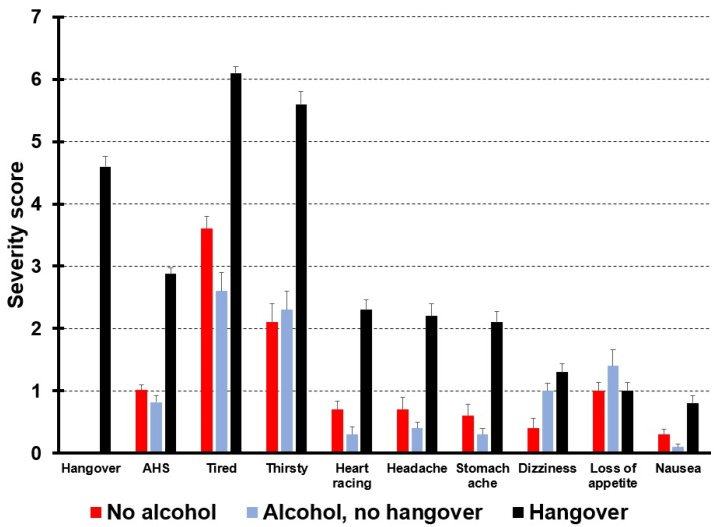
Presence and severity of symptoms related to alcohol hangover. Note: In contrast to the original AHS, scores range from 0 (absent) to 10 (extreme). Data from reference [19].

Hangover symptoms are also experienced when no alcohol is consumed. As a result, aggregate symptom scores may be greater than zero, even when no alcohol has been consumed. A recent study [19] compared hangover symptoms between subjects with and without a hangover and demonstrated that several symptoms are not unique to the hangover state but are also present without having a hangover or consuming alcohol. In this study, *n* = 299 subjects who were on holiday in Greece (mean (SD) age of 38.9 (11.0) years old) completed the AHS in the morning before walking the Samaria Gorge. *n* = 47 subjects consumed alcohol the evening before but reported having no hangover, *n* = 176 consumed alcohol and reported a hangover, and *n* = 76 consumed no alcohol and reported no hangover. Reported hangover symptoms from the three groups are depicted in Figure 3. 

First of all, these data again demonstrate that the mean (SD) AHS score of 2.9 (1.3) significantly underestimated the true hangover severity of 4.6 (2.1) among subjects with a hangover (*p* < 0.0001). Statistical comparisons of the individual hangover symptom scores between subjects who consumed no alcohol, those who consumed alcohol but reported no hangover, and drinkers with a hangover revealed that no significant differences between the groups were found for nausea and loss of appetite. Severity scores for headache did not significantly differ between drinkers with and without a hangover. The data in Figure 3 suggest that most symptoms that are attributed to the hangover state are always present, irrespective of alcohol consumption or having a hangover. It can be assumed that, when rating overall hangover severity via a 1-item scale, drinkers take into account that some symptoms may already be present on non-drinking days as well. For example, they may usually feel somewhat tired (although perhaps to a lesser extent than during the hangover state). This knowledge is then incorporated in their rating of hangover severity, which is more likely to reflect the difference/changes in symptom severity relative to a normal non-drinking day. Although the latter cannot be proven with the data at hand, we deem it to be a plausible hypothesis. Yet, hangover scales aggregate symptom scores without taking baseline symptom scores into account. As a result, a positive AHS hangover severity score can be obtained in subjects who reported to have no hangover after drinking alcohol (0.9) as well as in subjects who did not consume alcohol at all (1.0). In fact, their 1-item overall hangover severity score was zero. When relying solely on AHS scores, it would incorrectly be assumed that these subjects had a hangover. In this study, 95% of subjects who report no hangover via the 1-item overall hangover severity rating had an AHS score greater than zero and would be incorrectly labelled as having a hangover. When including these subjects in the dataset for statistical analysis, their AHS scores are, however, higher than those assessed with a 1-item severity score (i.e., zero), meaning that the AHS score overestimates the true overall hangover severity, producing false positives. When taken together, the findings that the severity of a true hangover tends to be underestimated (due the fact that not all of the items usually apply), while severity tends to be overestimated in the absence of a hangover, it seems that composite scores might be worse than 1-item overall ratings in differentiating between individuals with severe versus light hangover symptoms, by producing a tendency towards the middle. While it could theoretically be possible to try to identify false positives by the ratio of single-scale scores to overall ratings (even though this currently still remains to be tested), it would likely be impractical in most cases to have participants fill in an entire questionnaire, when a single-item overall hangover rating already provides the required information to a good, if not even better, degree. 

## 7. Day to Day Variability in the Presence and Severity of Hangover Symptoms

**Figure 4:**
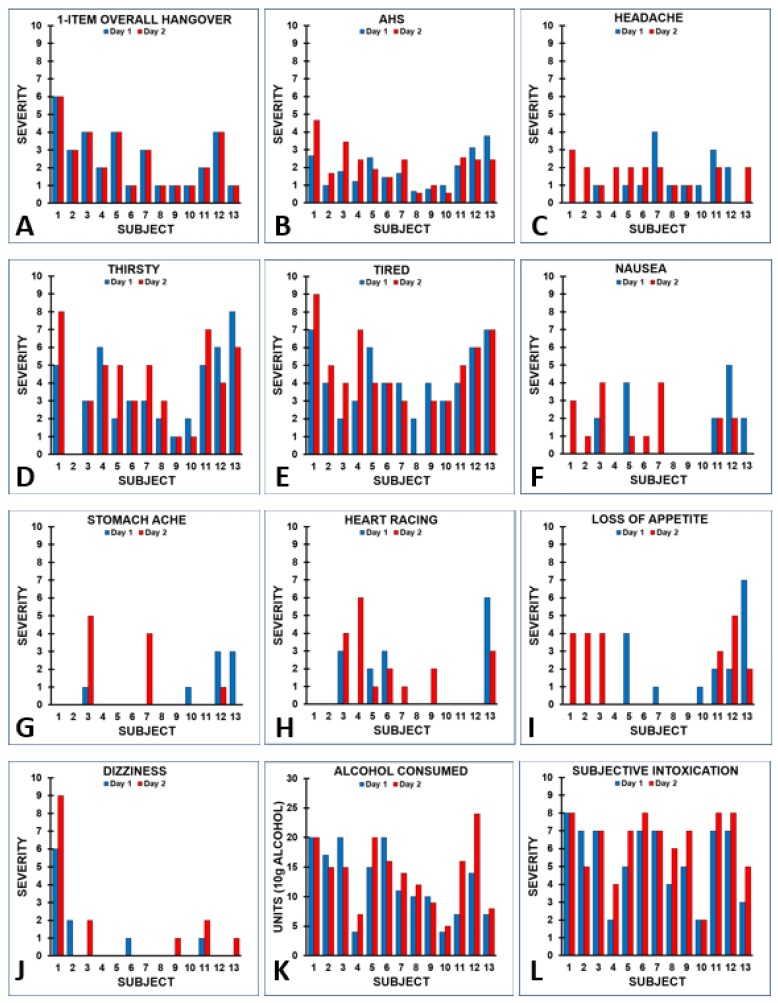
Level of subjective intoxication and alcohol consumption and corresponding next day hangover symptom severity reported on two different test days by the same subjects. Individual subject ratings for 1-item overall hangover severity (**A**), the AHS score (**B**), individual symptom scores (**C–J**), the amount of alcohol consumed the evening before having the hangover (**K**), and the corresponding level of subjective intoxication (**L**) are shown.

Van Wijk et al. [20] examined hangover severity of *n* = 22 students who were on a skiing holiday in Italy. The students experienced multiple hangovers during this period. Each morning at breakfast, subjects completed a modified AHS. The AHS included all nine symptoms, including a 1-item overall hangover severity score, but the severity scoring of items was modified to a range from 0 (absent) to 10 (extreme) In addition, past evening alcohol consumption was recorded and the level of subjective intoxication (i.e., drunkenness) was rated on a scale from 0 (absent) to 10 (extreme). For *n* = 13 subjects, it was possible to match two test days with identical hangover scores, as assessed with a 1-item overall hangover severity score. Several important observations were made when evaluating the data: First of all, the 1-item overall hangover severity score was different between subjects, but identical on the two test days (see Figure 4A). However, the AHS scores of the two test days showed considerable variability for some of the subjects (see Figure 4B), as the severity scores of individual hangover symptoms contributing to the aggregate AHS score differed between the two test days (see Figure 4C–J). In other words, despite having identical 1-item overall hangover severity scores, subjects reported considerable variability in individual symptom scores and overall AHS scores on the two test days. Finally, the data showed that having an identical 1-item overall hangover severity score does not necessarily imply that the same amount of alcohol was consumed, or that the corresponding level of reported intoxication was similar on the evening preceding the test day. Instead, subjects consumed different amounts of alcohol on both test days (see Figure 4K) and reported different levels of subjective intoxication (see Figure 4L). Notwithstanding this, their 1-item overall hangover severity scores on each test day were identical.

**Figure 5:**
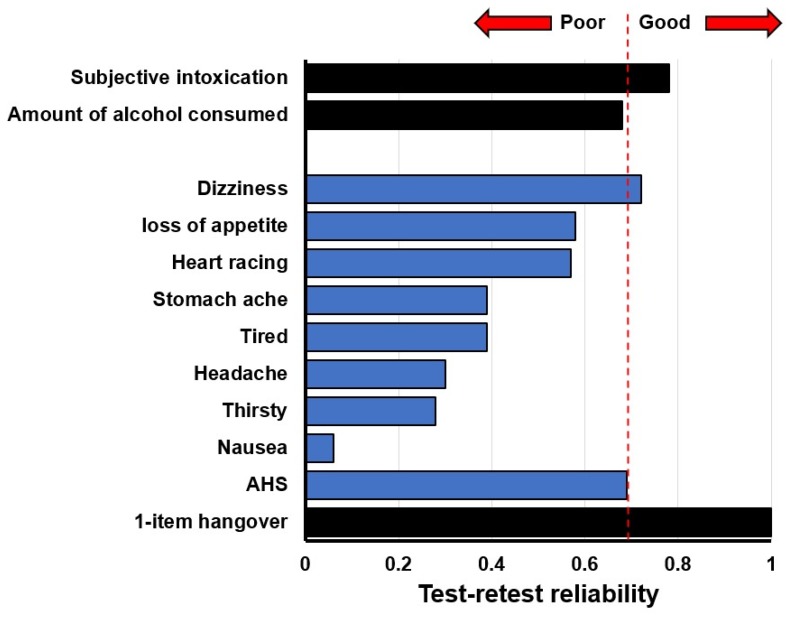
Test–retest reliability. Spearman’s correlations are shown. Higher scores suggest a better test–retest reliability. Bootstrapping (*n* = 10.000 samples, bias corrected 95% confidence interval) was applied to adjust correlations for the small sample size. An acceptable test–retest reliability is demonstrated if Spearman’s correlation > 0.7 [21].

Figure 5 shows the test–retest reliability of the AHS and its items. While the test–retest reliability of the 1-item overall hangover severity score was 1.0 (maximal, as test days had been selected to fulfil this criterion), the AHS test–retest reliability (0.69) was below the generally acceptable level of test–retest reliability of 0.7 [21]. The variability in individual hangover severity scores was greatest for headache, thirst and nausea, and none of the symptoms reached the acceptable limit of 0.7 for test–retest reliability, except for dizziness. Applying the more stringent Bland-Altman 95% limits of agreement method [22]—in which 95% of difference scores of day 1 and day 2 item or scale ratings should lie within the range of two standard deviations of the mean difference score to demonstrate agreement between the two assessments—revealed that no agreement was found for the symptoms of headache, heart racing, and loss of appetite. 

**Figure 6:**
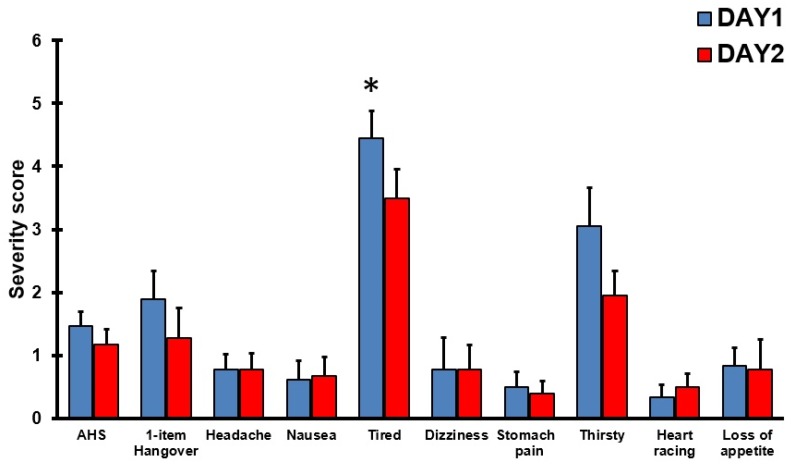
AHS and symptom severity scores for two days on which an equal amount of alcohol was consumed within subjects. Significant differences (*p* < 0.05) between the two days are indicated by an asterisk (*). Abbreviation: AHS = acute hangover scale.

An alternative way to look at the data is to select test days on which subjects consumed an identical amount of alcohol and then compare the presence and severity of AHS symptom scores. For *n* = 18 subjects, it was possible to match two test days with an identical amount of alcohol consumption. They consumed a mean (SD) of 11.6 (5.7) alcoholic drinks on these test days (range: 2 to 20 alcoholic drinks). Their AHS scores and individual symptom scores are summarized in Table 1 and Figure 6. Despite the fact that subjects consumed the same amount of alcohol on both test days, the data show considerable variability within subjects in both the presence and severity ratings on individual hangover symptoms, including the 1-item hangover severity score. 

Test-retest reliability for the AHS (r = 0.731, *p* = 0.001) was acceptable. With regard to individual symptoms, an acceptable test–retest reliability was, however, only found for the symptom of being tired (r = 0.775, *p* < 0.0001). No acceptable test–retest reliability was found for the ratings of overall hangover (r = 0.537, *p* = 0.022), and stomach pain (r = 0.569, *p* = 0.014). A poor test–retest reliability was found for being thirsty (r = 0.365, *p* = 0.136), dizziness (r = 0.351, *p* = 0.153), heart racing (r = 0.240, *p* = 0.337), headache (r = 0.186, *p* = 0.460), and nausea (r = 0.090, *p* = 0.723). The low test–retest reliabilities again confirm the fact that the presence and severity of hangover symptoms considerably varies between drinking occasions. In line with this, a recent study showed that there is great intraindividual variability in hangover severity scores between drinking occasions, even when the same amount of alcohol is consumed [23], and regression analyses demonstrated that the amount of consumed alcohol is usually not a strong predictor of hangover severity [24]. 

Table 1 further shows that the AHS scores on day 1 and day 2 are greater than zero for each subject. This could suggest that all of them experienced a hangover on both days. However, the 1-item overall hangover severity score demonstrated this to be incorrect, as six out of 18 subjects (33.3%) did not report having a hangover on day 1, and eleven subjects (61.1%) reported having no hangover on day 2. Taken together, relying solely on hangover symptom scales to assess the presence and severity of alcohol hangover will likely result in inaccurate results.

## 8. Should We Abandon the Use of Hangover Symptom Scales?

The fact that the outcomes of composite hangover scales do not appear to accurately reflect overall hangover severity does not imply that we should abandon their use altogether. In many cases, it is very relevant to assess the presence and severity of individual hangover symptoms. For example, if a company claims that treatment X is effective in reducing hangover headaches, it is highly relevant to assess “headache” severity, in addition to an assessment of overall hangover severity. As is evident from Section 2, there is a great variability in the presence and severity of individual hangover symptoms. It is also important to identify those symptoms that are most bothersome and impairing to subjects. For future research, it is therefore recommended to also assess individual hangover symptoms. This can be done by using one of the existing hangover scales, or simply by assessing individual symptoms of interest via symptom-specific 1-item severity scores. However, the judgement of the overall efficacy of a hangover treatment should preferably be based on a 1-item overall hangover severity rating, as this most likely incorporates all experienced symptoms and circumstances of the hangover state. As discussed in Section 5, symptoms experienced during hangover may also be experienced on non-hangover days. Therefore, is advisable to use difference scores for these individual symptoms when comparing their severity on a hangover day versus a no-hangover day to capture the “true” hangover effect. The latter does not apply for intervention studies, where a direct comparison of symptom scores between treatment and placebo should be made to evaluate a possible difference between the two hangover conditions.

Finally, it should be acknowledged that subjective ratings have sometimes been argued to be unreliable per se, thus mandating the assessment of biomarkers in order to have an objective assessment of hangover severity. Indeed, it would be very useful if such a biomarker would be discovered. Biomarkers related to alcohol metabolism and immune function may be promising candidates, but several lines of research have unfortunately not yet been able identified a suitable biomarker. Biomarkers can be assessed in various samples, including blood, saliva, hair, sweat, and stool, but, for practical use, volatiles in expired air would be ideal. However, the traditional breathalyzer readings reflecting ethanol concentrations are not useful, as BAC readings are often zero in the hangover state [25]. Alas, chemical compounds other than ethanol, which should ideally relate to hangover severity and/or functional impairments, would need to be detected by a breathalyzer in order to reliably indicate alcohol hangover. Notwithstanding this, the alcohol hangover is a complex state with many symptoms that may be experienced alone or in combination and may have differential severity and impact on daytime functioning. Given the currently available knowledge on the potential underlying mechanisms, it is still unclear whether a complex concept such as alcohol hangover can be accurately represented by a single biomarker. Further research investigating the suitability of potential volatile biomarkers in order to develop a breathalyzer for the hangover state is currently in progress.

## 9. Conclusions

Hangover symptoms can vary in their presence and severity between different drinking events in the same individual, and not all symptoms have equal impact in terms of impairment or being bothersome, regardless of their severity. Therefore, researchers should not rely solely on hangover symptom scales to assess overall hangover severity. Based on our reasoning, it is evident that composite, multi-item hangover symptom scales will likely underestimate severe hangovers and, at the same time, overestimate light hangovers, thus partly masking the true hangover effect. The resulting reduction in variance could hamper the ability to assess changes across study appointments or interventions and may thus have serious implications for the interpretation of study outcomes. Furthermore, the use of hangover symptom scales may also contribute to false positives, i.e., misidentifying subjects without a hangover as supposedly suffering from one. We propose that the most important, clinically meaningful endpoint for rating hangovers and the prevention or mitigation of hangovers by an effective product is the 1-item overall hangover severity score. This measurement allows the subject to assess the effect the condition is having on him or her, taking into account the symptoms being experienced, the severity of the symptoms being experienced and, most importantly, how the condition is impacting them in their activities of daily living and interactions with others, regardless of what the individual symptoms comprising his or her condition may be at the moment. The single greatest strength of the 1-item global assessment as a primary outcome measure is that it incorporates the subjects’ evaluation of the impact the specific subset of symptoms being experienced at that time, in place of and with greater subject-focused information value than the specific symptom-based sum score can provide. Thus, this single-score approach evaluates the entire constellation of the hangover state, regardless of the individual components contributing to it, in terms of presence, severity, and impact. 

Thus, the 1-item overall hangover severity rating represents a self-reported outcome instrument capable of measuring the severity of a condition (i.e., hangover) or the effect of a treatment in concordance with and incorporating all three concepts of an effective Patient-Reported Outcome Measure, namely, assessing the presence of symptoms, their effects on function, and their severity [13]. In addition, as secondary outcome measures (of efficacy), individual symptom presence and severity (or their impact) can be assessed using either hangover symptom scales, or individually. This will identify which individual symptoms are described by subjects as being the most bothersome during the alcohol hangover state.

## Figures and Tables

**Table 1:** AHS and symptom severity scores for two days on which an equal amount of alcohol was consumed by subjects.

Subject Number	Alcoholic Drinks	AHS Day 1	AHS Day 2	1-item HS Day1	1-item HS Day2
1	20	3.78	2.67	7	6
2	17	1.00	1.33	3	2
3	20	1.89	1.78	3	4
4	12	2.33	1.56	4	0
5	10	2.33	4.11	3	5
6	20	1.44	1.89	2	4
7	2	0.78	0.78	0	0
8	5	0.89	0.33	0	0
9	6	1.00	1.11	0	0
10	10	0.56	0.56	0	0
11	20	1.44	1.00	1	1
12	10	1.00	0.67	2	1
13	10	0.78	0.56	1	0
14	10	1.89	0.56	3	0
15	8	0.56	0.22	0	0
16	14	3.11	0.78	4	0
17	6	1.11	1.00	1	0
18	8	0.56	0.33	0	0
